# Patient-derived osteosarcoma cells are resistant to methotrexate

**DOI:** 10.1371/journal.pone.0184891

**Published:** 2017-09-21

**Authors:** Amanda dos Santos Cavalcanti, Walter Meohas, Gabriele de Oliveira Ribeiro, Ana Cristina de Sá Lopes, Sharareh Gholamin, Mostafa Razavi, Taís Hanae Kasai Brunswick, Amir Avan, João Antonio Matheus Guimarães, Maria Eugenia Leite Duarte, Suzana Assad Kahn

**Affiliations:** 1 Research Division, National Institute of Traumatology and Orthopaedics, Rio de Janeiro, Rio de Janeiro, Brazil; 2 Institute for Stem Cell Biology and Regenerative Medicine, Stanford School of Medicine, Stanford University, Stanford, California, United States of America; 3 National Center Structural Biology and Bio-imaging, Federal University of Rio de Janeiro, Rio de Janeiro, Rio de Janeiro, Brazil; 4 Institute of Biophysics Carlos Chagas Filho, Federal University of Rio de Janeiro, Rio de Janeiro, Rio de Janeiro, Brazil; 5 Molecular Medicine Group, Department of Modern Sciences and Technologies, School of Medicine, Mashhad University of Medical Sciences, Mashhad, Iran; Università degli Studi della Campania "Luigi Vanvitelli", ITALY

## Abstract

Osteosarcoma is the most common primary bone tumor in children and young adults. The median survival of osteosarcoma patients has not significantly improved since 1990, despite administration of different classes of chemotherapy agents, such as methotrexate, cisplatin and doxorubicin. Cancer stem cells (CSCs) are responsible for the resistance of osteosarcoma to chemotherapy and OCT4, SOX2 and SSEA4 have been used to identify CSCs in osteosarcoma. Here, we used low-passage patient-derived osteosarcoma cells and osteosarcoma cells directly isolated from patients before and after chemotherapy treatments to evaluate the effects of chemotherapy on stem cell markers expression. We demonstrate that primary osteosarcoma cells are resistant to methotrexate treatment and sensitive to cisplatin and doxorubicin *in vitro*. We also verified that cisplatin and doxorubicin reduce the expression of SOX2 and OCT4 in primary osteosarcoma cells whereas methotrexate does not alter SOX2 and OCT4 expression, however it increases SSEA4 expression in primary osteosarcoma cells. Finally, we found that, although the combination treatment cisplatin plus doxorubicin inhibited the *in vivo* growth of osteosarcoma cells in NOD-SCID gamma mice subcutaneously injected with SaOs2, the combination treatment cisplatin plus doxorubicin plus methotrexate did not inhibit the *in vivo* growth of these cells. These observations may provide an explanation for the poor response of osteosarcomas to chemotherapy and point to the need of reevaluating the therapeutic strategies for human osteosarcomas.

## Introduction

Osteosarcoma is the most common malignant bone tumor in children and young adults[1]. Despite chemotherapy interventions, the 5-year survival rates of osteosarcoma patients have remained at 50–80%[2] and the poor prognosis is due to the high incidence of metastasis and chemoresistance. Chemotherapy treatments that have shown activity against osteosarcoma include cisplatin, doxorubicin and high dose methotrexate[3, 4].

Although the origin of sarcomas remains unidentified, the high number of histopathological types and subtypes implies that sarcomas are a stem cell malignancy with multilineage differentiation capabilities that are caused by uncontrolled self-renewal[5, 6]. Identification of self-renewing cancer stem cells (CSCs), exclusively able to maintain long-term growth of hierarchically organized cancers[7], indicates that cancer therapies that target and extinguish CSCs may cure rather than just provisionally contain the disease[8]. CSCs may, therefore, be responsible for the resistance of osteosarcoma to chemotherapy. The elaboration of osteosarcoma stem cells (OSCs)-specific therapies, however, depends on the identification of OSCs and the molecular mechanisms that are crucial for their viability.

As prognostic evaluation of patients with osteosarcoma is still restricted to clinical considerations, molecular markers of tumor aggressiveness must be identified. Although osteosarcoma derives from the osteoblastic lineage, the nature of the cell of origin is still unclear. To date, markers such as CD133[9], CD117/Stro-1[6, 10], CBX3/ABCA5[11], OCT3/4[6], SOX2[12] and SSEA4[13] have been used to identify the OSCs. However, the mechanisms underlying the chemoresistance of osteosarcoma have not been revealed.

In this study, we analyzed stem cell markers expression in low-passage patient-derived osteosarcoma cells and in osteosarcoma cells directly isolated from patients before and after chemotherapy treatments. We demonstrate that primary osteosarcoma cells are resistant to methotrexate treatment and sensitive to cisplatin and doxorubicin *in vitro*. We also verified that cisplatin and doxorubicin reduce the expression of SOX2 and OCT4 in osteosarcoma cells. Methotrexate, on the other hand does not alter SOX2, OCT4 expression and increases SSEA4 expression in primary osteosarcoma cells. Understanding the effects of chemotherapy in osteosarcoma cells will contribute to the optimization of osteosarcoma treatments.

## Materials and methods

### Ethics statement

Osteosarcoma samples were obtained after written informed consent from each patient at the National Institute of Traumatology and Orthopaedics (INTO) in accordance with institutional board-certified protocols. The study “Isolamento e caracterização de células-tronco tumorais de Osteossarcoma” was approved by the local ethics committee (approval no.: 715.834; Ethics Committee on Research, INTO, Rio de Janeiro, Brazil).

### Patients and sample collection

Patients with histopathological evaluation of Osteosarcoma were included in this study (Table 1). Samples from patients who underwent diagnostic biopsy and resection surgery after chemotherapy (Table 2) were recovered from the center of the tumor mass using a 3mm T-Lok bone marrow biopsy needle (Angiotech), monitored by an X-ray image intensifier.

**Table 1:** Osteosarcoma patients’ characteristics, tumor topography, evolution, and surgical procedure.

Sample ID	Age (years)	Sex	Topography	Tumor evolution (months)	Surgical procedure
OS01	6	M	right distal femur	0.5	resection, amputation
OS02	15	M	right distal femur	6	resection, prosthesis
OS03	13	F	left proximal tibia	3	amputation
OS04	10	M	left proximal tibia	3	not performed
OS05	15	F	right distal femur	2	amputation
OS06	14	M	right distal ulna	6.5	amputation
OS07	10	F	left proximal tibia	3	amputation
OS08	10	M	right distal femur	1	amputation
OS09	12	F	right proximal tibia	not accessed	resection
OS10	22	M	right distal tibia	not acessed	amputation
OS11	16	M	left distal femur	not accessed	amputation
OS12	9	F	left distal femur	0.5	resection, prosthesis
OS13	18	M	left proximal tibia	not accessed	amputation
OS14	14	M	right distal femur	3	resection
OS15	16	M	left distal femur	4	amputation
OS16	16	M	right proximal tibia	3	amputation
OS17	9	M	right proximal humerus	5	not performed
OS18	15	F	left distal femur	4	amputation
OS19	57	F	left calcaneus	3	amputation
OS20	16	M	right proximal tibia	not accessed	amputation
OS21	22	M	right distal femur	not accessed	not performed
OS22	16	M	left distal femur	3	amputation
OS23	9	F	right distal femur	not accessed	amputation
OS24	14	M	left distal femur	2	not performed
OS25	18	M	left distal femur	not accessed	amputation
OS26	11	F	left distal femur	4	Amputation

M–male; F—female

**Table 2:** Histological tumor type, chemotherapeutic agents adopted, Huvos grade, alkaline phosphatase values (ALP), percentage of SOX2+ cells, relapse, presence of metastasis, and anatomic site of metastasis.

Sample ID	Histological Type	CH	Histological response (Huvos Grade)	ALP value above reference		% of SOX2^+^ cells >10%	Relapse	Metastasis			Death
				at diagnosis	after/during treatment			Anatomic Site	Months after diagnosis	Months after CH	
OS01	Conventional Central OS	Dox, Cis, MTX	II	2.4	2.7	yes	yes	lung	20.4	19.6	yes
OS02	Conventional Central OS	Dox, Cis	II	1.4	0.7	yes	yes	axillary lymph node	18.0	16.9	yes
OS03	Conventional Central OS	Dox, Cis, MTX, Ifo	II	0.6	0.5	no	no	lung	1.9	0.8	yes
OS04	Conventional Central OS	Dox, Cis, MTX	not performed	1.0	0.6	no	no	not applied	no	no	yes
OS05	Conventional Central OS	Dox, MTX,Cis, Eto, Ifo	II	1.0	0.5	no	no	not applied	no	no	no
OS06	Telangiectasic OS	Dox, Cis, Manitol, MTX	I	1.3	1.1	yes	no	lung	4.7	3.4	no
OS07	Conventional Central OS	Dox, Cis, MTX	IV	0.8	0.8	no	no	not applied	no	no	no
OS08	Conventional Central OS	Dox, Cis, MTX	II	0.6	1.0	yes	no	lung	7.5	6.8	no
OS09	Conventional Central OS	Dox, Cis, MTX, Eto, Ifo	II	1.7	1.0	no	no	lung, brain	11.3	10.2	no
OS10	Conventional Central OS	Dox, Cis, Ifo	not performed	0.8	0.8	no	no	lung	0.5	before CH	yes
OS11	Telangiectasic OS	Dox, Cis, MTX	IV	0.2	0.1	no	no	not applied	no	no	no
OS12	Conventional Central OS	Dox, Cis, MTX, ETO, IFO	III	2.2	0.5	no	no	lung	13.5	12.1	no
OS13	Conventional Central OS	Dox, Cis, MTX	III	0.4	0.7	no	no	not applied	no	no	no
OS14	Pleomorphic Central OS	Cis, Dox, Ifo, Eto	not accessed	0.7	0.4	no	no	lung	when first seen	before CH	no
OS15	Telangiectasic OS	Dox, Cis, MTX	II	0.7	0.8	yes	no	lung	3.5	5.8	no
OS16	Conventional Central OS	Dox, MTX,Cis, Eto, Ifo	II	1.0	0.8	no	no	lung	8.9	7.6	no
OS17	Conventional Central OS	Dox, Cis, Ifo	not performed	25.0	not performed	yes	no	lung, axillary lymph node	10.9	10.7	yes
OS18	Conventional Central OS	Cis, Dox, MTX	I	not performed	not performed	no	no	not applied	no	no	no
OS19	Conventional Central OS	Dox, Cis	not performed	1.2	0.6	no	no	not applied	no	no	no
OS20	Telangiectasic OS	Dox, Cis, MTX	I	0.7	not accessed	no	no	lung	2.9	2.6	yes
OS21	Chondroblastic OS	Dox, Cis, Ifo	not performed	0.8	0.6	no	no	lung	30	29	no
OS22	Conventional Central OS	Dox, Cis, MTX	not performed	1.5	1.0	no	no	not applied	no	no	no
OS23	Conventional Central OS	Cis, Dox, MTX	III	0.4	0.5	no	no	lung	0.8	before CH	no
OS24	Conventional Central OS	Dox, Cis, MTX, Eto	not performed	not accessed	0.6	no	no	lung, costal arch	0.9	0.3	yes
OS25	Conventional Central OS	Dox, Cis, Ifo	II	0.7	0.4	yes	no	lung	12.4	6.8	no
OS26	Conventional Central OS	Dox, MTX,CIS, Manitol, ETO, IFO	III	0.7	0.8	yes	no	lung	2.7	4.2	yes

OS, osteosarcoma; Dox, doxorubicin; CIS, cisplatin; MTX, methotrexate; ETO, etoposide; IFO, ifosfamide; CH, chemotherapy.

### Chemicals and reagents

Anti-CD34-PE, anti-CD15-PE, anti-CD184-PE, anti-SSEA4-V450 and the isotype controls were purchased from BD Biosciences. Anti-CD133/2-PE was from Miltenyi Biotec. Anti-Sox2 was from Millipore. 3-(4,5-Dimethylthiazol-2-yl)-2,5-diphenyltetrazolium-bromide (MTT), dimethylsulfoxide (DMSO), 4-6-diamino-2-phenylindole (DAPI), cis-diammineplatinum-II-dichloride, doxorubicin hydrochloride, methotrexate hydrate and Fluoromount Aqueous Mounting Medium were from Sigma Aldrich. TrypLE and ACK lysing buffer were from Life Technologies. EnVision™ FLEX, High pH and EnVision FLEX Target Retrieval Solution, High pH were obtained from Dako. Protease inhibitor cocktail tablets were from Roche. SuperSignal West Pico Chemoluminescent Substrate was from Thermo Scientific.Bergisch Gladbach, Germany.

### Processing of tumor samples and cell culture

Tumor samples were readily collected and washed in a solution of 0.6% glucose with Antibiotic-Antimycotic in phosphate buffer saline (PBS) to remove blood clots. Then, they were mechanically dissociated in a solution containing Dulbecco’s Modified Eagles Medium/Nutrient Mixture F-12 Ham (DMEM-F12), 2.5% collagenase-II and Antibiotic-Antimycotic at 37°C for 12h. The suspension was washed twice with PBS-glucose solution and incubated for 5min with ACK lysing buffer on ice to eliminate blood cells. After washing, the pellet was filtered through a 70μm cell strainer. Single cells were immediately used to perform experiments or cultured in DMEM-F12 supplemented with 10% fetal bovine serum (FBS) and 0.1X Antibiotic-Antimycotic and grown at 37°C, 5% CO_2_.

### Lentiviral transduction of osteosarcoma cells

The pCDH-CMV-MCS-EF1-puro HIV-based lentiviral vector (Systems Bioscience, USA) construct contains an ubiquitin promoter driving the expression of a luciferase-eGFP fusion product[14]. The luciferase gene is the Luc2 (pgl4) version (Promega, USA). The eGFP portion derives from the pIRES2-eGFP plasmid (Becton Dickinson, USA). Lentiviral production and concentration was accomplished using standard protocols. SaOs2 were transduced for 12 h at 37°C, 5% CO_2_, with lentivirus containing 6 μg/mL polybrene. After 24 h, cells were washed repeatedly to remove extracellular lentivirus. Cell sorting of eGFP-positive SaOs2 cells was performed on a BD FACS Aria (Becton Dickinson, USA).

### Animal care

A total of 24 females 4–6 week old NOD.Cg-Prkdcscid Il2rgtm1Wjl/SzJ (NOD-SCID gamma) mice (body weights, 15–20g) were used. Animals received water *ad libitum* and were fed with irradiated rodent diet. Mice were housed in specific pathogen-free conditions (filtered rack, ALESCO^®^, Brazil) under 12-hour light/dark cycles at an animal facility at the National Institute of Traumatology and Orthopaedics (INTO) in Rio de Janeiro, Brazil.

All animal handling, surveillance, and experimentation was performed in accordance with and approval from the Ethic Commission on Animal Use of the National Institute of Traumatology and Orthopaedics (protocol no.: CEUA INTO 001/2014).

### *In vivo* transplantation of osteosarcoma cells

SaOs2 cells were transduced with a GFP and luciferase encoding lentivirus and double sorted to obtain a pure luciferase-expressing population. A tumorigenic dose of 2 x 10^6^ cells (suspended in 0.1 mL) was injected subcutaneously into the flanks of 4–6 week old NOD-SCID gamma mice. Tumor formation was followed by bioluminescence imaging on IVIS spectrum (Caliper Life Science) and quantified with Live Image 4 software. D-luciferin (firefly) potassium salt solution (Biosynth) was prepared (16 mg/mL) and injected intra-peritoneally (0.139 g luciferin per kilogram body weight). Total flux (photons per second) values were obtained by imaging mice until peak radiance was achieved and quantified with Live Image 4.0 software. Once tumor masses were detected, mice were randomized in three groups (i) control (without treatment), (ii) cisplatin in combination with doxorubicin, and (iii) a combination of cisplatin, doxorubicin, and methotrexate. Cisplatin (10 mg/Kg), doxorubicin (10 mg/Kg) and methotrexate (5 mg/Kg) were delivered intraperitoneally once a week for 60 days. The animals were observed daily. Severe tumor burden (more than 20 mm in diameter), difficulty breathing and prostration were considered as early endpoint. At the end of the treatment, after which mice were euthanized with CO2, tumors were resected for cell isolation (please refer to Processing of Tumor Samples).

### MTT citotoxicity assays

Osteosarcoma cells were seeded with 10% FBS DMEM-F12 medium in 96-well plates and cultured for 24h. Cells were then treated with chemotherapeutic drugs (doxorubicin, cisplatin and methotrexate) or with the vehicles (5% glucose solution, 0.9% sodium chloride and 0.1M sodium hydroxide, respectively) for 72h. Viable cells were quantified by the MTT cytotoxicity assay as previously described[15]. The cell viability was measured at each drug concentration as the ratio of absorbance at 560nm, relative to vehicle-treated cells.

### Flow cytometry cnalysis

Osteosarcoma cells were detached by gentle enzymatic treatment with TrypLE for 5min, 37°C. Then, they were washed in 1X PBS and centrifuged at 0.3 rcf for 5min, 4°C. After that, pellet was resuspended in a solution of 0.5% bovine serum albumin (BSA) in PBS and stained with CD133/2-PE (BD Biosciences), CD15-PE, or SSEA4-V450 (Miltenyi Biotech) for 30min, 4°C. Appropriate isotype controls were also used. DAPI was used to assess the cell population viability. Flow cytometry analysis was performed on a FACS Aria-II (BD Biosciences) and using CellQuest Pro software (BD Biosciences). To determine the effects of the chemotherapeutic agents, cells were treated with 100 μM of each agent.

### Immunohistochemistry

Primary osteosarcoma samples were formalin-buffered fixed and paraffin-embedded. Sections (5μm) were routinely processed. After deparafinization under 70°C in a dry incubator, tissue sections were subjected to antigen retrieval with EnVision FLEX Target Retrieval Solution, High pH. Immunostaining was performed with EnVision™ FLEX, High pH, following manufacturer’s instructions using rabbit anti-Sox2 for 40min at room temperature. The sections were counterstained with haematoxylin and examined by light microscopy (Nikon Eclipse TS100). Image processing was done using Adobe Photoshop-CS5 software.

### Western blotting

Single cells were washed twice with cold PBS and processed as previously described[16]. Briefly, cells were lysed in 1% NP40, 1% TritonX-100, 1% sodium deoxycholate, 10mmol/L Tris-HCl pH 7.5, 100mmol/L NaCl, and 0.1% sodium dodecyl sulfate (SDS), 5mM EDTA, supplemented with protease inhibitor. Membranes were blocked with 5% nonfat milk in tris-buffered saline with 0.1% Tween-20 (TBS-T) for 30min, incubated with rabbit anti-Sox2 overnight 4°C, and incubated with peroxidase-conjugated secondary antibody for 1h at room temperature. Bands were obtained after exposing the membranes to an X-ray film using the SuperSignal West Pico Chemoluminescent Substrate and then analyzed by densitometric scanning and quantified using ImageJ software.

### Immunocytochemistry

Immunocytochemistry analysis was performed as previously described[15]. Briefly, cells were fixed with 4% paraformaldehyde (PFA) in PBS for 15min, washed with PBS and incubated with 5%BSA for 30min. Cells were incubated with anti-Sox2 in 1% BSA overnight at 4°C, washed and incubated with secondary antibodies for 2h. Cells were then stained with DAPI and mounted with Fluoromount Aqueous Mounting Medium. Negative controls were performed with rabbit IgG. Imaging was performed with a confocal microscope (Leica-TCS-SP5) equipped with a 63xNA 1.40-oil-immersion objective. Image processing was done using ImageJ software.

### Statistical analysis

Statistical analysis tests are specified in figure legends. The level of significance was set at p<0.05, and results are shown as mean ± SD of at least three independent experiments performed with at least triplicates per condition. For *in vivo* experiments, 8 mice per cohort were used. Samples or animals were not excluded from the analysis. Statistical analyses were carried out with Prism 6.0 software (GraphPad).

## Results

### SOX2 is expressed in osteosarcoma tissue and cells directly isolated from patients

**Fig 1:**
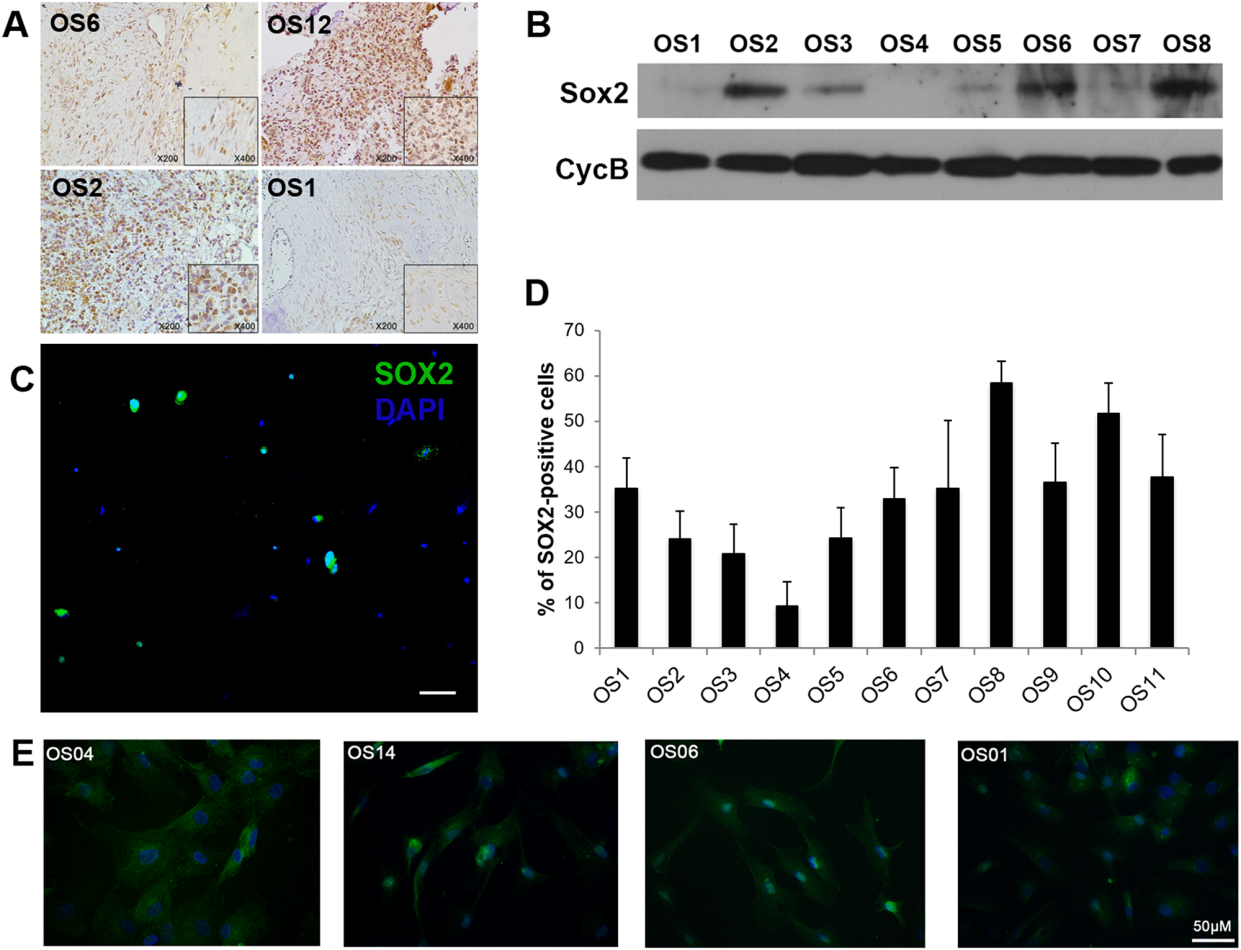
Analysis of SOX2 expression in human osteosarcoma tissue and cells directly isolated from patients. **A**. Representative immunohistochemistry images showing expression of SOX2 in osteosarcoma tissues from four patients (OS1, OS2, OS6 and OS12). **B**. Western blot analysis of SOX2 expression in primary tumor cells directly isolated from the tumor sites of eight osteosarcoma patients. Cyclophilin B was used as loading control. **C**. Representative immunocytochemistry image showing SOX2 expression in cells (OS6) immediately after tumor tissue dissociation. Scale bar, 100μm. **D**. Quantification of SOX2-positive cells from immunofluorescence in freshly dissociated samples from 11 osteosarcoma patients. **E**. Immunofluorescence staining of osteoprotegerin in patient-derived osteosarcoma cells in culture. Scale bar = 50 μm. OPG, osteoprotegerin.

Previous research has shown that SOX2 maintains self-renewal of tumor initiating cells in osteosarcoma cell lines[12]. Immunoperoxidase staining in paraffin-embedded tumor tissues revealed the expression of SOX2 in all osteosarcoma tissues analyzed (Fig 1A).

As the *in vitro* environment is known to alter the markers expression in cells in culture[17], we investigated SOX2 expression in cells directly isolated from the tumor site of osteosarcoma patients, without having contact to the culture microenvironment. The levels of SOX2 expression in cells immediately after tumor tissue dissociation from eight different patients were accessed by Western blot and we found that osteosarcoma samples express different levels of SOX2 (Fig 1B). To determine the percentage of SOX2-positive cells in tumor samples, we performed immunocytochemistry assays (Fig 1C) in tumor cells isolated from 11 patients and found that the percentage of SOX2-positive cells varies from 10% to 60% (Fig 1D). The patient-derived cells (Fig 1E) and cell lines (SaOs2 and MG-63, S1 Fig) express osteoprotegerin, a marker of osteoblastic differentiation.

### Methotrexate does not reduce the viability of patient-derived osteosarcoma cells

The main chemotherapeutic agents that have been adopted to treat osteosarcoma patients are cisplatin, doxorubicin and methotrexate[3, 4]. Among the 26 patients included in this study, 19 received chemotherapy treatments with cisplatin, doxorubicin and methotrexate (Table 2).

**Fig 2:**
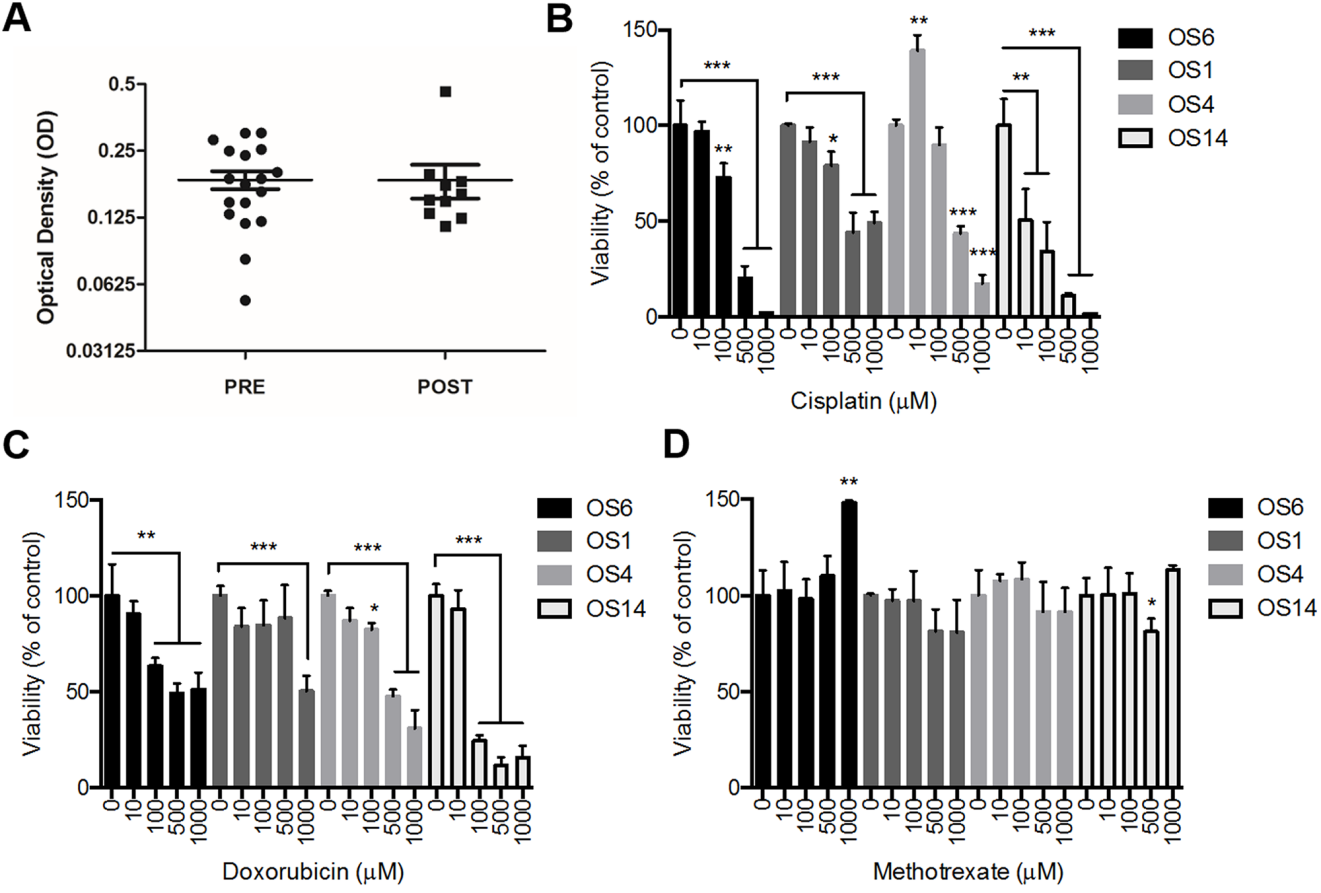
Effect of chemotherapeutic agents in osteosarcoma cells viability. **A**. MTT analysis of osteosarcoma cells isolated from 18 PRE samples (from 18 different patients) and 10 POST samples (from 8 different patients, as samples from OS2 and OS3 were collected at two different time-points after chemotherapy). **B-D**. MTT analysis of osteosarcoma cells isolated from four patients (OS1, OS4, OS6 and OS14) and treated with vehicle or **B**. cisplatin, **C**. doxorubicin, or **D**. methotrexate for 72h. * *P* < 0.05, ** *P* < 0.01, *** *P* < 0.001, Mann-Whitney U test.

To compare the viability between osteosarcoma cells isolated from patients before (PRE) and after (POST) chemotherapy treatments, we performed MTT assays with low-passage tumor cells derived from pre-chemotherapy surgeries (PRE)and post-chemotherapy surgeries (POST) and we found that these two groups of cells presented similar viability profiles (Fig 2A). We have collected 18 PRE samples (from 18 different patients) and 10 POST samples (from 8 different patients, as samples from OS2 and OS3 were collected at two different time-points after chemotherapy).

In order to analyze the effect of each chemotherapeutic agent in osteosarcoma cells viability, we treated low-passage PRE cells from four different patients (OS1, OS4, OS6 and OS14), and SaOs2 cells (S2 Fig) with increasing concentrations of cisplatin (Fig 2B), doxorubicin (Fig 2C) or methotrexate (Fig 2D). Cisplatin (Fig 2B) and doxorubicin (Fig 2C) significantly reduced the viability of all primary osteosarcoma cells and SaOs2. Methotrexate, on the other hand, marginally reduced OS14 viability at 500μM, increased the viability of OS6 at 1000μM (Fig 2D), and did not affect the viability of SaOs2 at 24h (S2 Fig). These data indicate that low-passage human osteosarcoma cells are sensitive to cisplatin and doxorubicin, but are resistant to methotrexate treatment *in vitro*.

### Methotrexate does not reduce SOX2 and OCT4 expression in patient-derived osteosarcoma cells

**Fig 3:**
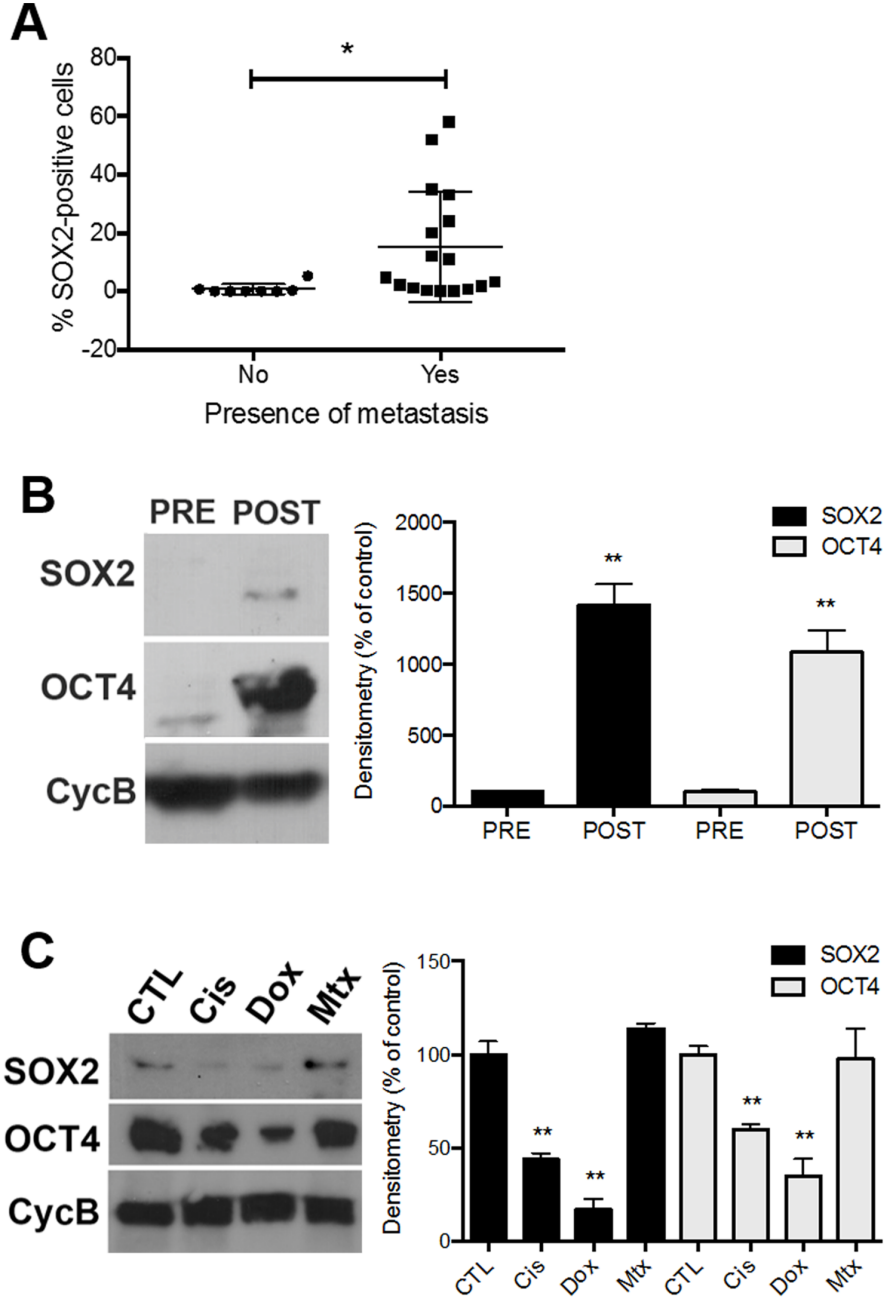
Analysis of SOX2 and OCT4 expression in osteosarcoma cells treated with chemotherapy agents. **A**. Correlation between presence of metastasis in osteosarcoma patients and SOX2 expression in cells derived from their tumors. **B**. Western blot analysis of SOX2 and OCT4 expression in primary tumor cells directly isolated from osteosarcoma patients (OS1 and OS6) before (PRE) and after (POST) chemotherapy treatments. Cyclophilin B was used as loading control. **C**. Western blot analysis of SOX2 and OCT4 expression in primary tumor cells (OS1 and OS9) after *in vitro* treatment with cisplatin, doxorubicin or methotrexate. * *P* < 0.05, ** *P* < 0.01, Mann-Whitney U test.

The outcome for patients with nonmetastatic disease at presentation has greatly improved, with the 5-year event-free survival (EFS) ranging between 60–70%[18]. However, the EFS for patients with metastatic disease, usually located in the lung, ranges between 11%[19] and 47%[20]. We have previously verified that SOX2 is expressed in osteosarcoma tissue and cells directly isolated from patients (Fig 1). We then analyzed the correlation between the percentage of SOX2-positive cells from tumor samples isolated from 25 patients and the presence of metastasis in these patients (Table 1). We found that patients that presented with metastasis also presented higher levels of SOX2-positive tumor cells than patients that did not present with metastasis (Fig 3A).

To analyze whether the chemotherapy treatments alter the levels of stem cell markers expression in osteosarcoma cells, we performed Western blot assays with osteosarcoma cells directly isolated from the tumor sites before (PRE) and after (POST) chemotherapy cycles (Fig 3B) and low-passage primary osteosarcoma cells treated with chemotherapeutic agents *in vitro* (Fig 3B). Osteosarcoma primary cells isolated after chemotherapy treatments (POST) express higher levels of SOX2 and OCT4 than cells isolated from the same patients (OS1 and OS6) before chemotherapy (PRE) (Fig 3B).

To assess the role of each chemotherapy agent in SOX2 and OCT4 expression, low passage osteosarcoma cells from two patients (OS1 and OS9) were treated with 100 μM cisplatin (Cis), doxorubicin (Dox) or methotrexate (Mtx). Cisplatin and doxorubicin consistently reduce SOX2 and OCT4 expression in patient-derived osteosarcoma cells (Fig 3C). Methotrexate, on the other hand, does not alter SOX2 and OCT4 expression in these cells.

### Methotrexate increases SSEA4 expression in patient-derived osteosarcoma cells and does not inhibit osteosarcoma growth *in vivo*

CD133/prominin-1[21], a cancer stem cell marker, SSEA4 (stage-specific embryonic antigen 4)[22], a glycoprotein expressed early in embryonic development and in pluripotent stem cells, and SSEA1/CD15 (stage-specific embryonic antigen 1)[23] have been identified in osteosarcoma cells[13, 21–23]. However, the effect of chemotherapy on the expression of these markers in osteosarcoma cells has not been reported.

**Fig 4:**
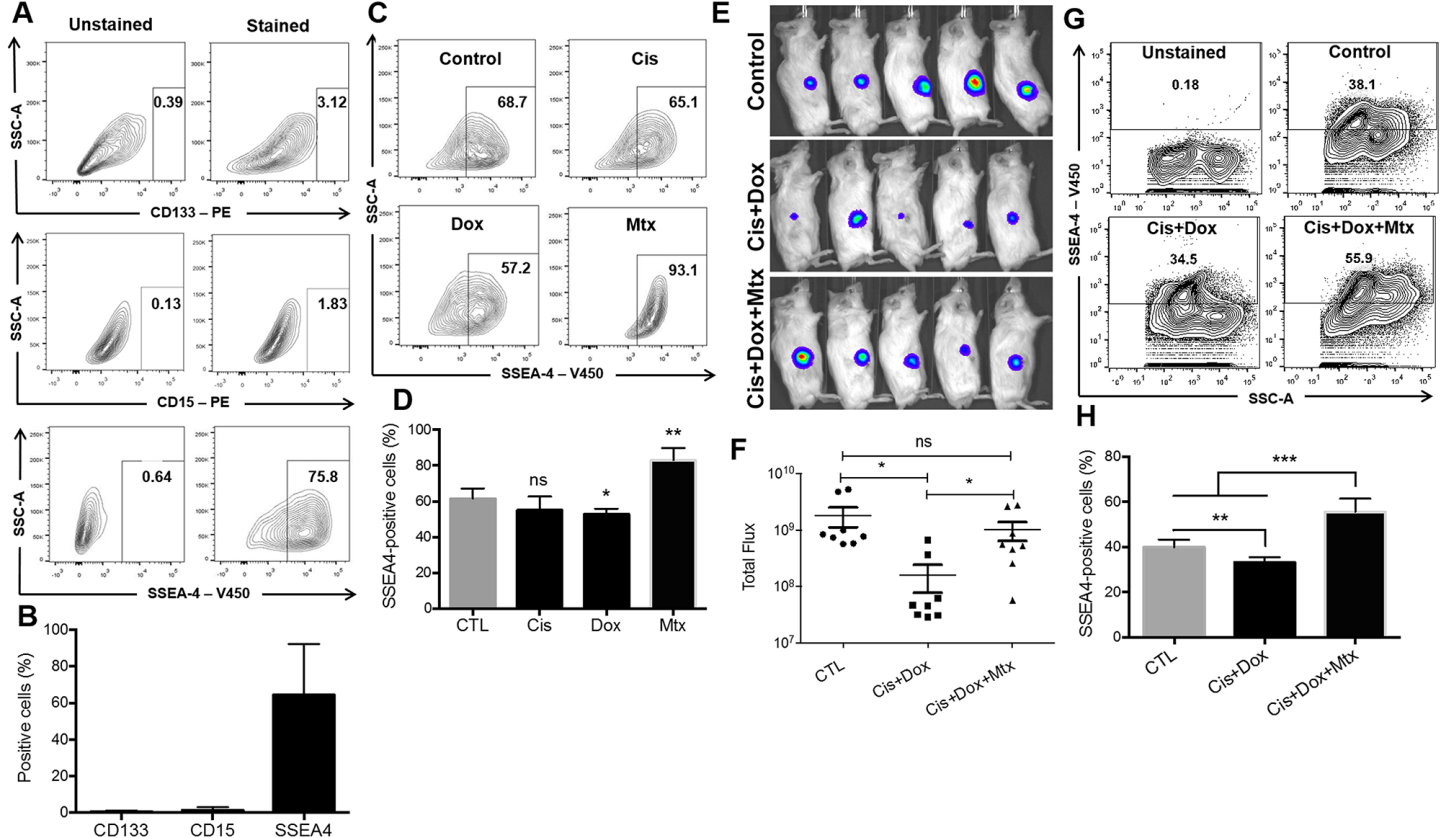
Effect of chemotherapeutic agents in SSEA4 expression in osteosarcoma cells. **A, B**. Flow cytometry analysis (**A**) and quantification (**B**) of CD133, CD15 and SSEA4 expression in patient-derived osteosarcoma cells. **C, D**. Flow cytometry analysis (**C**) and quantification (**D**) of SSEA4 expression in primary osteosarcoma cells treated with cisplatin, doxorubicin or methotrexate. **E**. Bioluminescent imaging of mice injected with luciferase-expressing SaOs2 osteosarcoma cells and treated with cisplatin and doxorubicin (Cis+Dox), or cisplatin, doxorubicin and methotrexate (Cis+Dox+Mtx). **F**. Quantification of total flux from tumors. **G, H**. Flow cytometry analysis (**G**) and quantification (**H**) of SSEA4 expression in osteosarcoma cells isolated from tumors treated with cisplatin and doxorubicin (Cis+Dox), or cisplatin, doxorubicin and methotrexate (Cis+Dox+Mtx). * *P* < 0.05, ** *P* < 0.01, *** *P* < 0.001, Mann-Whitney U test.

The cell-surface phenotype of low-passage osteosarcoma cells was characterized using flow cytometry (Fig 4). In accordance to previous studies[21, 23], we found that only a small fraction of patient-derived osteosarcoma cells express CD133 and CD15 in their surface (Fig 4A and 4B). On the other hand, ~70% of primary osteosarcoma cells are SSEA4-positive (Fig 4A and 4B). Of note, SaOs2 cells do not express SSEA4 *in vitro* (S3 Fig). To further assess the role of chemotherapy in stem cell markers expression, we treated primary osteosarcoma cells with 100μM cisplatin (Cis), doxorubicin (Dox) or methotrexate (Mtx) and analyzed SSEA4 expression. Cisplatin and doxorubicin did not alter SSEA4 expression (Fig 4C and 4D). Methotrexate, on the other hand, increases SSEA4 expression in the surface of osteosarcoma cells (Fig 4C and 4D).

To assess the role of methotrexate in osteosarcoma growth *in vivo*, SaOs2 cells were engineered for constitutive expression of GFP and luciferase and subcutaneously injected into the flank of immune compromised NOD.Cg-*Prkdc*^scid^ *Il2rg*^tm1Wjl^/SzJ (NSG) mice. After confirmation of tumor growth by bioluminescent imaging, mice were treated with the chemotherapy agents (see Methods section). The combination treatment cisplatin and doxorubicin (Cis+Dox, n = 8) inhibited the *in vivo* growth of osteosarcoma cells (Fig 4E and 4F) compared to control (n = 8) and Cis+Dox+Mtx groups (n = 8). However, the combination treatment cisplatin, doxorubicin and methotrexate (Cis+Dox+Mtx) did not inhibit the *in vivo* growth of osteosarcoma cells (Fig 4E and 4F). These tumors were then harvested, dissociated and analyzed for SSEA4 expression. Osteosarcoma cells isolated from mice treated with Cis+Dox presented lower levels of SSEA4 expression, as compared to control (Fig 4F and 4G). However, osteosarcoma cells isolated from mice treated with Cis+Dox+Mtx presented increased levels of SSEA4 expression (Fig 4F and 4G).

Together, our findings indicate that patient-derived osteosarcoma cells are sensitive to cisplatin and doxorubicin, but resistant to methotrexate. Furthermore, methotrexate increases SSEA4 expression in patient-derived osteosarcoma cells and does not inhibit tumor growth *in vivo*.

## Discussion

In this study, we investigated the effect of chemotherapy in osteosarcoma viability and stem cell markers expression. SOX2 and OCT4 are more expressed in tumor cells isolated from osteosarcoma patients after chemotherapy treatments, compared to tumor cells isolated from the same patients before chemotherapy. Additionally, we found that osteosarcoma cells isolated from different patients express variable levels of SOX2 expression and different percentage of SOX2-positive cells. Finally, we suggest that low-passage human osteosarcoma primary cells are sensitive to cisplatin and doxorubicin, but are resistant to methotrexate.

SRY (sex determining region Y)-box 2 (SOX2) is a member of the large SOX gene family, comprising transcription factors recognized as keys in the regulation of developmental processes and cell type specification[24]. The main member SOX2 plays crucial roles in the maintenance of cell pluripotency and self-renewal in both embryonic stem cells[25] and in induced pluripotent stem cells[26]. Recently it has also been reported an oncogenic role of SOX2 by regulating osteosarcoma stem cells self-renewal[12]. Moreover, miR-126 was described as an inhibitor of osteosarcoma proliferation, migration and invasion by suppressing SOX2 expression[27]. Here, we analyzed the levels of SOX2 expression in samples immediately after osteosarcoma tissue dissociation by immunohistochemistry (Fig 1A), Western-blot (Figs 1B and 2B), and immunofluorescence (Fig 1C and 1D). We found that SOX2 expression in cells derived from tumors correlate with the presence of metastasis in osteosarcoma patients (Fig 3A), and that tumor cells freshly isolated from patients after chemotherapy treatments (POST) express higher levels of SOX2 and OCT4 than tumor cells isolated before chemotherapy (PRE) (Fig 3B).

Markers can be uninformative *in vitro*, as there is an ongoing cell population selection that precludes the ability to make inferences about the existence of hierarchy[17]. Hence, their expression and usefulness in OSCs identification may be missed if evaluated in culture versus freshly dissociated tumors. Here, we developed and used patient-derived osteosarcoma cells for the characterization of chemotherapy agents currently adopted in the clinic. We analyzed cells directly isolated from tumor tissues from osteosarcoma patients, without having contact with the culture environment. To reduce the possibility of having contamination with another cell types, the samples were removed from the center of the tumor, monitored by real-time X-Ray, and treated with ACK lysis buffer to eliminate red blood cells. To assure the osteoblastic identity of patient-derived osteosarcoma cells, the primary cultures were stained for osteoprotegerin (Fig 1E), a marker of osteoblastic phenotype[28]. It has already been shown that primary osteosarcoma cells preserve some aspects of the normal osteoblast phenotype[29].

Patient-derived tumor samples express different levels of SOX2 (Fig 1), suggesting that the cancer stem cell population burden varies across osteosarcoma patients. Furthermore, higher percentage of SOX2-positive tumor cells positively correlates with the presence of metastasis in osteosarcoma patients (Fig 3A), suggesting that SOX2 may be a potential prognostic marker for metastasis in osteosarcoma patients. Although surrounding normal tissue and red blood cells were eliminated from the tumor mass, cells from the immune system, endothelial cells and osteoclasts for example may still be present in the cellular suspension. Therefore, a study that extensively addresses the role of each cell type (including tumor-associated macrophages, dendritic cells, CD8+ T cells, CD4+ T cells, Tregs, NK cells) in the tumor bulk *in vivo* and human data analysis would help understand how each cell type present in the tumor mass responds to tumor therapies. Moreover, recent advances in single-cell technologies have opened new avenues to characterize the intra-tumor cellular heterogeneity, and, ultimately, guide diagnosis and treatment.

CD133 was recently described as a marker for osteosarcoma stem cells[21]. In accordance to previous studies[21, 30], we found that only a small fraction of patient-derived osteosarcoma cells express CD133 in their surface (Fig 4A). Therefore, in this study, SOX2 (Figs 1 and 3), OCT4 (Fig 2B and 2C), and SSEA4 (Fig 4) have been used to identify CSCs in osteosarcoma. In fact, osteosarcoma stem cells have been shown to overexpress self-renewal and pluripotency markers, such as OCT4, SOX2 and NANOG, and drug transporters, such as the Mitoxantrone resistance protein (MXR/BCRP1/ABCG2), which has been shown to participate in the multi-drug resistance of osteosarcoma[31–33]. The upregulation of stem cell and drug resistance markers in tumor samples may indicate the undifferentiated state of the tumor, resistance to chemotherapeutic agents and consequently unfavorable prognosis of osteosarcoma patients. Interestingly, although SaOs2 cells are positive for SSEA4 *in vivo* (Fig 4G and 4H), they do not express SSEA4 in culture (S3 Fig). In fact, Zhang *et al*[13] have shown that SaOs2 is a “mature”, lineage-committed, cell line and therefore negative for SSEA4. They also show that partially differentiated SSEA4-negative osteosarcoma cells dedifferentiate to regenerate SSEA4-positive tumor initiating cells. Moreover, adherent cells in culture usually present a more differentiated profile, while *in vivo* inoculation restores the stemness of the putative tumor initiating cells. In this study, we show that primary tumor cells directly isolated from osteosarcoma patients after (POST) chemotherapy treatments express higher levels of SOX2 and OCT4 than primary tumor cells directly isolated from osteosarcoma patients before (PRE) chemotherapy treatments (Fig 2B). While cisplatin and doxorubicin *in vitro* treatment reduced SOX2 and OCT4 expression in primary tumor cells, methotrexate did not affect SOX2 and OCT4 expression (Fig 2C). In fact, methotrexate increased the expression of SSEA4 in osteosarcoma cells both *in vitro* (Fig 4C) and *in vivo* (Fig 4G). The contribution of NANOG and ABCG2 in patient-derived osteosarcoma cells resistance to methotrexate still needs to be clarified. Although we have collect a total of 26 primary tumor samples (Tables 1 and 2), some samples yield very low cell numbers, restricting the amount of data that can be extracted from these samples. Therefore, Fig 2A does not include all the 26 patients.

CSCs are highly resistant to current cancer treatments[8, 34]. This suggests that many cancer therapies, while killing the majority of tumor cells, may finally fail because they do not eliminate the CSCs, which survive to regenerate the tumor mass. Therefore, our findings showing that osteosarcoma cells isolated from patients after chemotherapy express higher levels of stem cell markers might be reflecting the resistance of osteosarcoma cells to current chemotherapy treatments and a selection of the CSCs that reestablish the tumor bulk. Another interpretation is that the chemotherapeutic agents might be increasing osteosarcoma stem cells proliferation.

Here, we show that cisplatin (Fig 2B) and doxorubicin (Fig 2C) reduce the viability of primary osteosarcoma cells in a dose-dependent manner. Methotrexate, on the other hand, was not able to reduce the viability of these cells (Fig 2D). Instead, high doses of methotrexate increased the viability of OS6 (Fig 2D). Osteosarcoma resistance to methotrexate can be originated by different adaptive molecular mechanisms, including modifications of drug targets, metabolic pathways, drug influx / efflux, and activation of savage pathways. Multi-drug resistance (MDR) is normally a consequence of overexpression of membrane-active transporters responsible for drug extrusion out of the cell[35]. Methotrexate is not able to passively cross cell membranes, needing specific transporters for cell internalization. Therefore, mutations or reduced expression of these transporters, such as folate carrier (RFC), or increased expression of dihydrofolate reductase (DHFR) reduce drug membrane transport and consequently lead to resistance to the drug[36]. Although methotrexate is not generally adopted separately to osteosarcoma patients, as is normally administered as part of a combination therapy with cisplatin and doxorubicin, this result points to the need of further analysis on the effect of chemotherapy agents on osteosarcoma cells. Therefore, characterizing the intracellular pathways associated with chemotherapeutic agents and how they affect self-renewal and tumor resistance may help designing novel anti-cancer drugs that effectively reduce tumor relapse in osteosarcoma patients.

In this research, we provide evidence for the existence of CSCs in human primary osteosarcomas in patients before (PRE) and after (POST) chemotherapy treatments. We propose, for the first time, that POST cells express higher levels of stem cells markers than PRE cells from the same patients. We also suggest that primary osteosarcoma cells are resistant to methotrexate treatment and sensitive to cisplatin and doxorubicin *in vitro*. These observations may provide an explanation for the poor response of osteosarcomas to chemotherapy and point to the need of reevaluating the therapeutic strategies for human osteosarcomas.

## Supporting information

S1 Fig Immunofluorescence staining of osteoprotegerin in SaOs2 and MG-63 cell lines. Scale bar = 50 μm. OPG, osteoprotegerin.
(TIF)

S2 Fig MTT analysis of osteosarcoma cell lines SaOs2 and MG-63 treated with doxorubicin (Dox), cisplatin (Cis), or methotrexate (Mtx) for 24h. *** P < 0.001, One-way ANOVA followed by Tukey's post hoc analysis.
(TIF)

S3 Fig Flow cytometry analysis of SSEA4 expression in SaOs2 cells in culture. (TIF)

S1 ARIVE Checklist ARRIVE Guidelines Checklist. (DOCX)
