# Supplementary figures and images for: Patient-derived osteosarcoma cells are resistant to methotrexate
Source: PLoS One. 2017 Sep 21;12(9):e0184891. doi: 10.1371/journal.pone.0184891 (PMC5608286; doi:10.1371/journal.pone.0184891)

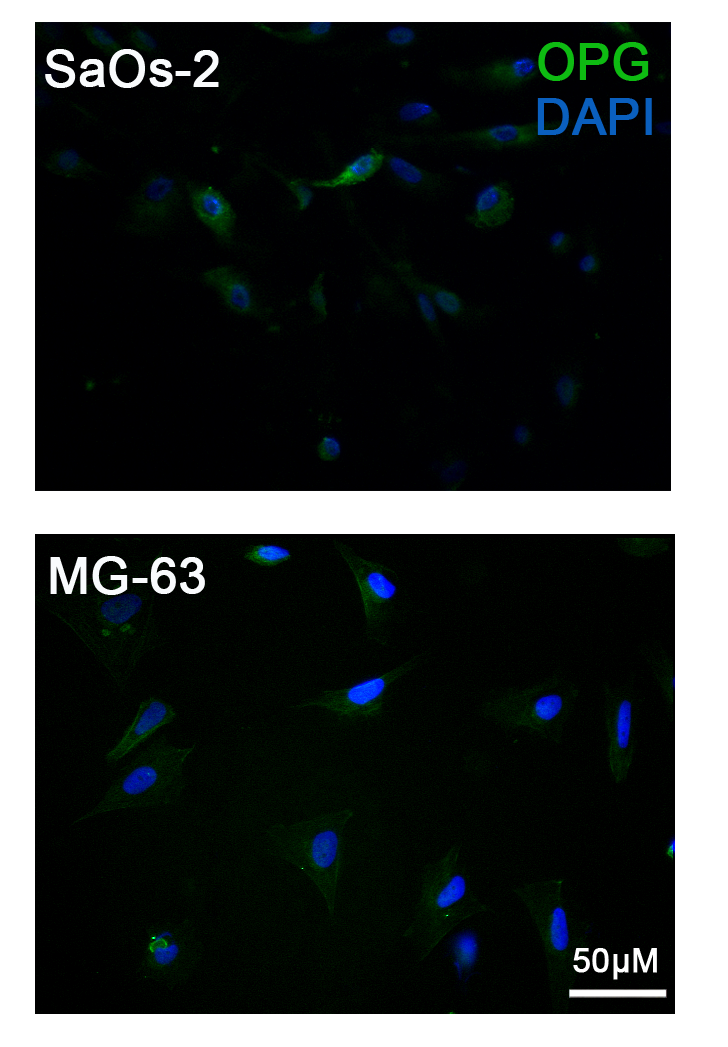

Supplement: S1 Fig — Scale bar = 50 μm. OPG, osteoprotegerin. (TIF) [file pone.0184891.s001.tif]

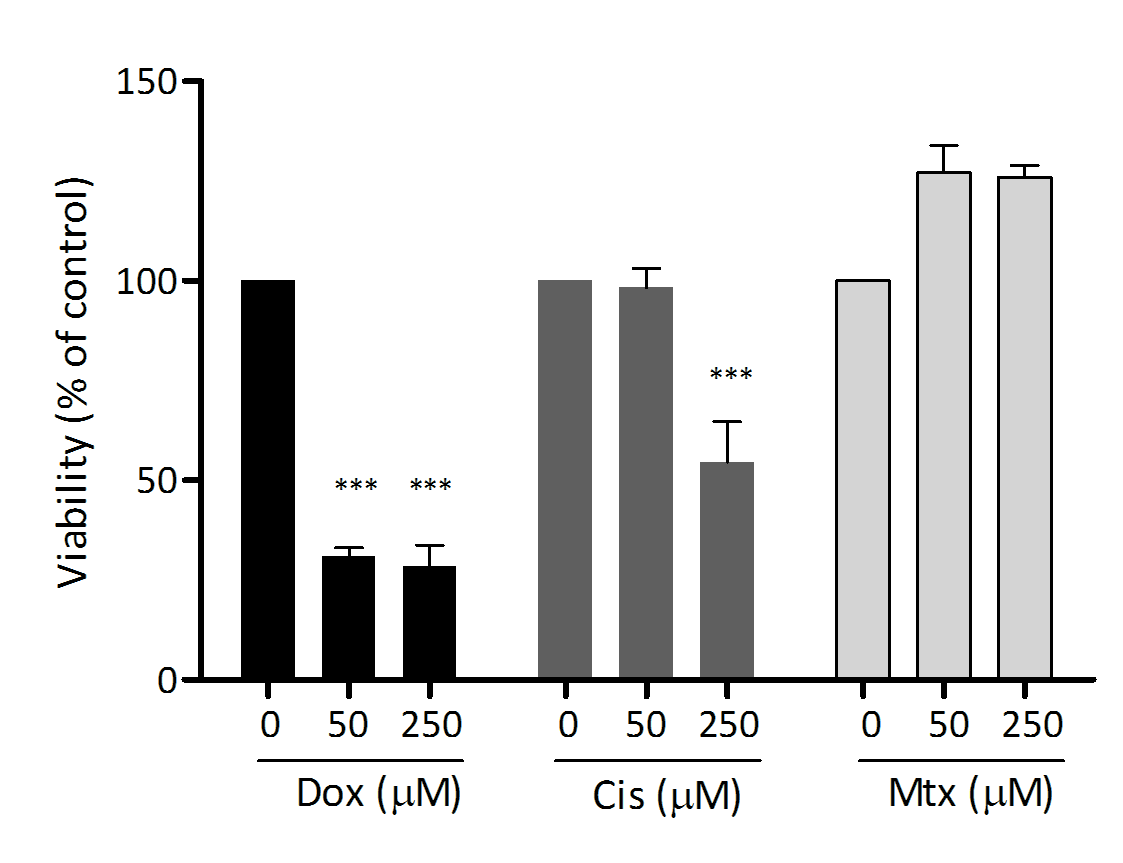

Supplement: S2 Fig — *** P < 0.001, One-way ANOVA followed by Tukey's post hoc analysis. (TIF) [file pone.0184891.s002.tif]

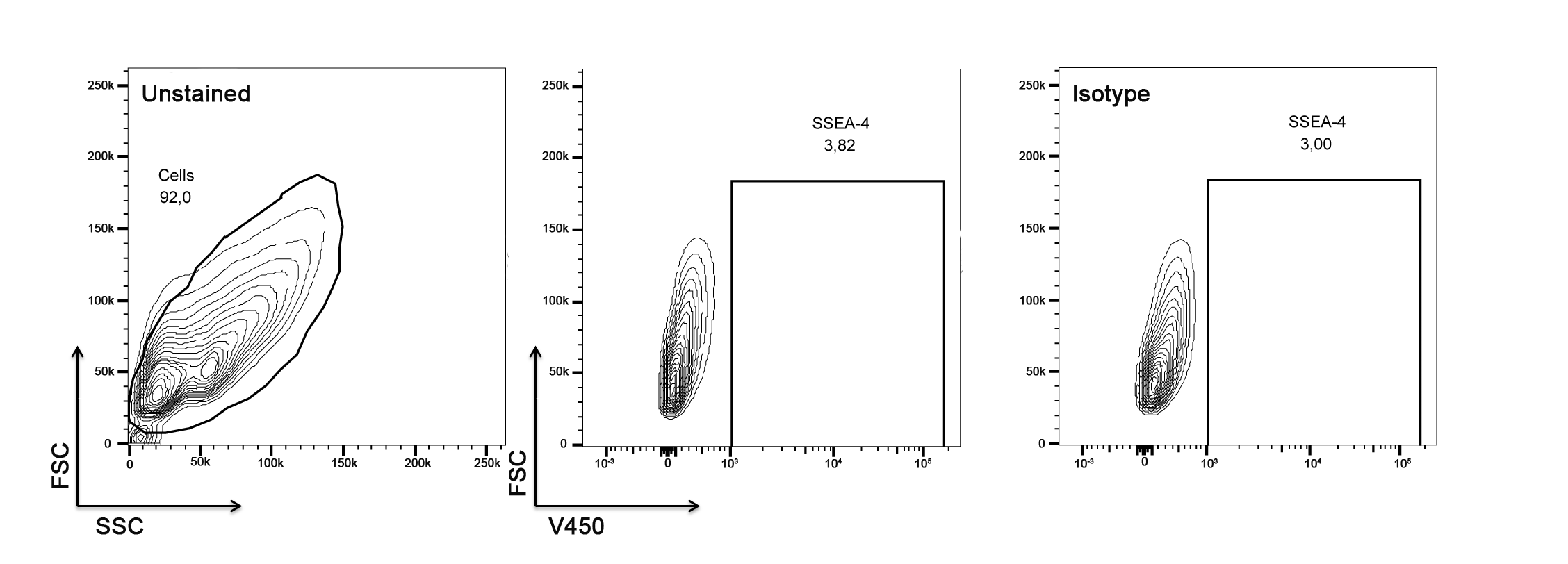

Supplement: S3 Fig — (TIF) [file pone.0184891.s003.tif]
